# Extracellular Matrix Regulates UNC-6 (Netrin) Axon Guidance by Controlling the Direction of Intracellular UNC-40 (DCC) Outgrowth Activity

**DOI:** 10.1371/journal.pone.0097258

**Published:** 2014-05-13

**Authors:** Yong Yang, Won Suk Lee, Xia Tang, William G. Wadsworth

**Affiliations:** Department of Pathology, Rutgers Robert Wood Johnson Medical School, Piscataway, New Jersey, United States of America; McGill University, Canada

## Abstract

How extracellular molecules influence the direction of axon guidance is poorly understood. The HSN axon of *Caenorhabditis elegans* is guided towards a ventral source of secreted UNC-6 (netrin). The axon’s outgrowth response to UNC-6 is mediated by the UNC-40 (DCC) receptor. We have proposed that in response to the UNC-6 molecule the direction of UNC-40-mediated axon outgrowth is stochastically determined. The direction of guidance is controlled by asymmetric cues, including the gradient of UNC-6, that regulate the probability that UNC-40-mediated axon outgrowth is directed on average, over time, in a specific direction. Here we provide genetic evidence that a specialized extracellular matrix, which lies ventral to the HSN cell body, regulates the probability that UNC-40-mediated axon outgrowth will be directed ventrally towards the matrix. We show that mutations that disrupt the function of proteins associated with this matrix, UNC-52 (perlecan), UNC-112 (kindlin), VAB-19 (Kank), and UNC-97 (PINCH), decrease the probability of UNC-40-mediated axon outgrowth in the ventral direction, while increasing the probability of outgrowth in the anterior and posterior directions. Other results suggest that INA-1 (α integrin) and MIG-15 (NIK kinase) signaling mediate the response in HSN. Although the AVM axon also migrates through this matrix, the mutations have little effect on the direction of AVM axon outgrowth, indicating that responses to the matrix are cell-specific. Together, these results suggest that an extracellular matrix can regulate the direction of UNC-6 guidance by increasing the probability that UNC-40-mediated axon outgrowth activity will be oriented in a specific direction.

## Introduction

**Figure 1:**
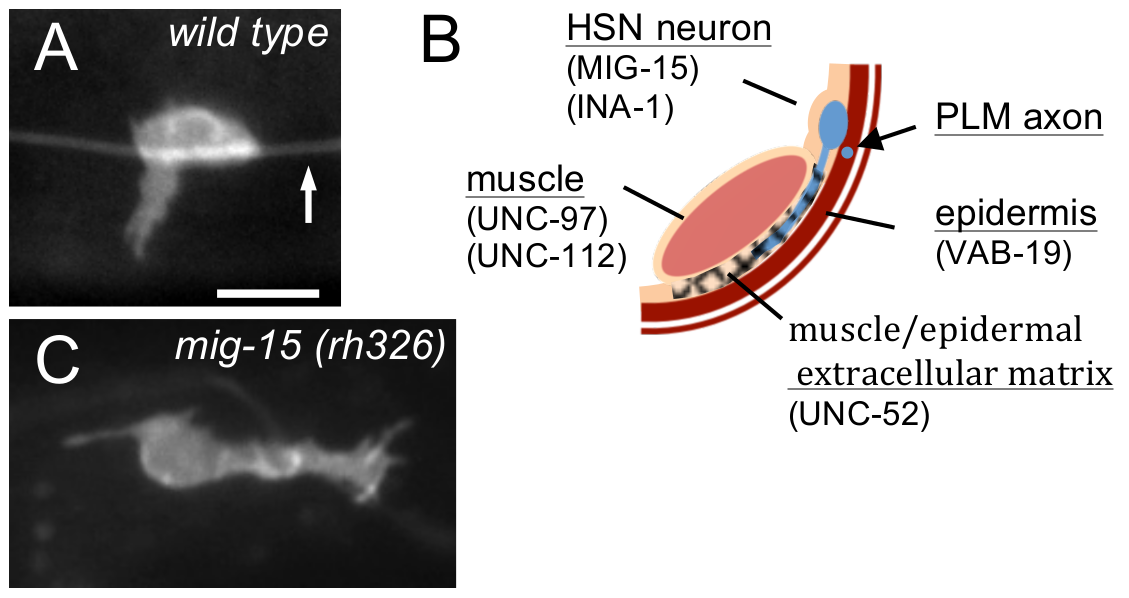
HSN Axon Outgrowth is directed into the Muscle/Epidermal Extracellular Matrix. (A) Photomicrograph of a wild-type L3 stage animal showing ventral HSN axon protrusion from the cell body. Arrow indicates the PLM axon. Scale bar: 5 µm. (B) Schematic diagram of the right ventral body wall showing the relative position of HSN axon outgrowth. The HSN neurites invade the extracellular matrix that lies between the longitudinal muscle cells and the epidermis. The longitudinal axon of the mechanosensory PLM neuron is enveloped by the epidermis and has specialized attachments to the epidermis [32]. The reported locations where the molecules considered in this study function are indicated. (C) Photomicrograph of a wild-type L3 stage *mig-15* animal showing posterior HSN axon protrusion from the cell body. In mutants, the axon often protrudes anteriorly or posteriorly along the dorsal side of the muscle/epidermis interface.

Axons are guided by cues emanating from the surrounding extracellular environment. A major challenge has been to understand how extracellular cues specify the direction of axon guidance. The formation of the *Caenorhabditis elegans* HSN axon is a model for studying axon guidance. Details of HSN axon development were described in Adler et al., 2006. Immediately after hatching, the neuron extends short neurites in random directions from the cell body. However, the neurites soon become restricted to the ventral side of the cell where a ventral leading edge forms. This leading edge is just dorsal of a band of longitudinal body-wall muscle cells (Figure 1A, B). At this interface the ventral surface of HSN pauses and expands until, abruptly, neurites extend and invade the specialized basement membrane that separates the muscles and the epidermis. This matrix contains specialized structures that allow the muscle cells to attach to the epidermis. In fact, it is somewhat surprising that circumferentially migrating axons transverse this specialized complex matrix instead of taking a seemingly easier route along the matrix at the internal side of the muscle [1].

The major extracellular cue that directs HSN axon formation and guidance is UNC-6 (netrin) [2]–[5]. Recent evidence suggests that the response mediated by the UNC-40 (DCC) receptor to the UNC-6 *molecule* is stochastic; axon outgrowth activity is induced and is randomly oriented in different directions [6], [7]. A second UNC-40-mediated response is to the UNC-6 *gradient*. This separate response increases the probability that the randomly directed axon outgrowth activity induced by the first signal will be oriented towards the ventral UNC-6 source. In wild-type animals, the separate effects of the two signals are not observed since the UNC-6 gradient increases to nearly 100% the probability that UNC-40-mediated axon outgrowth activity will be directed ventrally. The evidence for the separate signals comes from the phenotypes caused by the *unc-40(ur304)* mutation, and by mutations that disrupt the function of UNC-53, a cytoskeletal binding protein [6], [7]. The *unc-40(ur304)* mutation encodes an UNC-40 variant, UNC-40 (A1056V), that is proposed to partly mimics the confirmation of UNC-40 when UNC-6-ligated [7]. UNC-53 is thought to repress UNC-6-independent UNC-40 signaling [6]. In the *unc-40* and *unc-53* mutants, randomly directed UNC-40-mediated axon outgrowth activity is induced in the *unc-6(−)* background and the axon has a probability of protruding in each direction. In the *unc-6(+)* background, UNC-40-mediated axon outgrowth activity and axon protrusion is ventral. The interpretation is that in the mutants an UNC-40 signal induces stochastically directed axon outgrowth activity, which in the *unc-6(−)* background cannot be oriented towards the ventral UNC-6 source.

The conclusion that a separate process orients the outgrowth activity raises the possibility that external asymmetric factors, besides the UNC-6 gradient, could help regulate the probability that the UNC-40-mediated outgrowth activity will be oriented ventrally. Although an extracellular molecule might not induce the UNC-40-mediated axon outgrowth activity itself, the asymmetric distribution of that molecule could provide directional information for UNC-6 guidance by altering the probability of UNC-40-mediated axon outgrowth at each side of the neuron. Previous evidence suggests that the Wnt EGL-20 functions in this manner [6]. EGL-20 is required to prevent UNC-40-mediated axon outgrowth activity in the anterior and posterior directions. This observation indicates that the UNC-6 gradient by itself is not sufficient to prevent the direction of axon outgrowth activity from fluctuating.

Our conclusion that the direction is stochastically determined in response to UNC-6 raises the question of how the neuron decides the direction of UNC-6 guidance. In wild-type animals, the probability that the HSN axon will protrude ventrally is very high. However in some mutants, such as *egl-20*, the probability that UNC-40 will asymmetrically localize to the ventral side of the neuron is comparatively lower, whereas the probability of localization to the anterior and posterior sides is higher. Yet, the HSN axon is still guided to the ventral midline by UNC-6. This observation is evidence that the direction of UNC-40 axon outgrowth activity, and the outward force it generates, fluctuates during axon outgrowth [6]. A biased random walk is a mathematical formulization of a path consisting of a succession of movements in which the direction at each step is randomly determined with a consistent bias for a specific direction. Despite the fact that there is a probability that the axon will be directed anteriorly or posteriorly, the UNC-6 gradient creates an overall bias for ventral outgrowth so that on average, over time, the axon is directed ventrally. Another characteristic of a random walk is that the mean square displacement (msd) will increase only linearly with time, whereas the msd increases quadratically with time for straight-line motion. Based on this property, it would be expected that on average it would take longer for an axon to develop in a mutant than in a wild-type animal since the same amount of force could not move an extension as far in the same amount of time. In fact, in mutants, such as *egl-20,* the formation of a morphologically mature axon is delayed in an UNC-40-dependent manner [6].

We reasoned that the extracellular matrix that is just ventral of the HSN cell body could, along with the gradients of UNC-6 and EGL-20 wnt, affect the probability that the UNC-40-mediated axon outgrowth activity directs the axon ventrally. In part because the basement membrane molecule UNC-52 (perlecan) is strongly associated with this specific matrix [8], [9] and it has been shown to affect UNC-6 guidance [10]. We were also intrigued by similarities between HSN axon development and the invasion of the anchor cell into the vulval epithelium. In both cases UNC-6 directs a cell extension through a basement membrane and in both cases UNC-40 becomes localized within a cell to the side that protrudes into the basement membrane [11], [12].

In the anchor cell, the integrin heterodimer INA-1/PAT-3 is required to localize UNC-40 (DCC) to the invasive membrane [13]. In neurons, INA-1/PAT-3 is known to mediate a response to UNC-6 through interactions with MIG-15, a homologue of Nck-interacting kinase (NIK) [14]–[16]. Intriguingly, when axonal growth cones reach the muscle/epidermis matrix in *mig-15* mutants they abnormally spread and will sometimes elongate in the anterior and posterior directions, *i.e.,* the invasion of this matrix is disrupted [15]. This phenotype is comparable to the HSN anterior and posterior axon protrusion phenotype observed in Wnt/PCP mutants. We therefore hypothesized that INA-1/MIG-15 signaling might affect HSN guidance by similarly regulating the localization of UNC-40 towards the muscle/epidermis matrix.

Here we provide evidence that the muscle/epidermis extracellular matrix and integrin/MIG-15 signaling regulate UNC-6 axon guidance by helping create a directional bias for UNC-40-mediated outgrowth activity in HSN. We show that mutations that alter the muscle/epidermal extracellular matrix do not prevent UNC-6 from inducing an asymmetric localization of UNC-40 within the neuron. However, they do affect the probability of where UNC-40 asymmetrically localizes within the neuron. In comparison to wild-type animals, the probability of UNC-40 localizing to the ventral side of the neuron decreases, while the probability of UNC-40 localizing at the anterior or posterior sides of the neuron increases. This phenotype is also observed in *ina-1* and *mig-15* mutants, and we confirm that *mig-15* functions cell autonomously in HSN. Axon development is consistent with the mutations causing the direction of UNC-40-mediated axon outgrowth activity to fluctuate within the neuron. As predicted by the biased random walk model, the axon may protrude from the cell body in the anterior or posterior direction but it nearly always reaches the ventral midline. Furthermore, there is an UNC-40-dependent delay in axon development. We also note that the effect the muscle/epidermis extracellular matrix has on the direction of axon outgrowth is cell specific; similar outgrowth patterns are not observed for the AVM axon, which also is guided into and through this matrix by UNC-6 and UNC-40 signaling.

## Materials and Methods

### Strains

Strains were handled at 20°C unless stated otherwise by using standard methods (Brenner, 1974). A Bristol strain N2 was used as wild type strain. The following strains were used in this paper: **LGI**, *unc-40(e1430), zdIs5[mec-4::GFP]*; **LGII**, *unc-52(e998), unc-52(e444)*, *vab-19(e1036)*; **LGIII**, *ina-1(gm39)*, *ina-1(gm144);* **LGIV,** *unc-5(e53), unc-112(r367), kyIs262 [unc-86::myr-GFP; odr-1::dsRed], zdIs13[tph-1::GFP];* **LGX**, *unc-6(ev400)*, *unc-97(su110), mig-15(rh80), mig-15(rh326).*

Transgenes maintained as extrachromosomal arrays included: *kyEx1212 [unc-86::unc-40-GFP;ord-1::dsRed], urEx386[unc-86::MIG-15(forward), unc-86::MIG-15(reverse), myo-3::mCherry], gmEx593[unc-86::MIG-15(genomic), myo-2::GFP].*

### Transgenic Vectors

For cell-specific mig-15 knock-down experiment, PCR from *C. elegans* cDNA library (Invitrogen) was performed to make genetic vectors containing mig-15 sequence using forward (AAAACCGCTAGCGGATCCATGTCGTCATCAGGACTCGAC) and reverse (ACCAGTGGTACCTTACCTAGGCCAATTTGTCAACCCTGGCTTA) primers. The PCR product was cut with NheI and KpnI and inserted into pSM [*unc-86::unc-40::gfp*] vector (Adler et al., 2006) for forward direction. The constructed *unc-86::mig-15* (forward) vector was cut by either NheI and AgeI or XmaI and AvrII, and those two fragments were combined to make *unc-86::mig-15* (reverse). Both vectors containing forward and reverse direction of *mig-15* sequences are injected with *myo-3::mCherry* as a co-injection marker.

### Analysis of the Timing of HSN Axon Outgrowth

HSN neurons were visualized using an integrated *unc-86::myrGFP* transgenic strain, *kyIs262*. Synchronized populations were obtained by allowing eggs to hatch overnight in M9 buffer without food. The resulting L1, L2, and L3 nematodes larvae were mounted on a 5% agarose pad. Larval staging was determined by the gonad cell number. An outgrowth was defined as a protrusion if it extended a distance greater than the length of two cell bodies. A precocious phenotype was scored in the L1 or L2 stage if either HSN in an animal showed a predominate axon. Only one HSN was counted as having an axon per animal. The ventral neurite was scored if it had any ventral filopodia or a leading edge formation during middle L3 stage, and the most prevalent direction of growth was noted. The standard error of proportion was calculated for this purpose. Images were taken using epifluorescent microscopy with a Zeiss 63× water immersion objective.

### Analysis of the Axon Phenotype in L4 Stage Animal

For analysis of the AVM axon protrusion phenotype, L4 stage larvae were mounted on a 5% agarose pad. The AVM axon was visualized in L4 stage larvae expressing the *zdIs5[mec-4::GFP]* transgene. Axons were scored as having an anterior protrusion if the axon traveled laterally more than three cell body lengths from the cell body. Axons were scored as having dorsal or posterior protrusion if the axon traveled dorsally or posteriorly for a distance greater than two cell body lengths from the cell body. The AVM was considered multipolar if more than one process, greater than one cell body length, was observed.

HSN neurons were visualized using an integrated *unc-86::myrGFP* transgenic strain, *kyIs262*. L4 stage larvae were mounted on a 5% agarose pad. An anterior protrusion was scored if the axon extended from the anterior side of the cell body for a distance greater than the length of three cell bodies. A dorsal or posterior protrusion was scored if the axon extended dorsally or posteriorly for a distance greater than two cell body lengths. The HSN was considered multipolar if more than one process extended a length greater than one cell body. Ventral parallel HSN axon growth was counted if two axons grow ventrally in parallel after crossing into the muscle/epidermis interface. Images were taken using epifluorescent microscopy with a Zeiss 40× objective.

### Analysis of the UNC-40::GFP Localization in L2 Stage Animal

Staging was determined by the gonad cell number and gonad size. For analysis of UNC-40::GFP localization, L2 stage larvae expressing the *kyEx1212[unc-86::unc-40::GFP; odr-1::dsRed]* transgene were mounted in M9 buffer with 10 mM levamisole. Images were taken using epifluorescent microscopy with a Zeiss 63× water immersion objective. To determine the HSN position and the stage of the animal, all animals were imaged by differential interference contrast (DIC) microscopy as well. The UNC-40::GFP localization was determined by measuring the average intensity under lines drawn along the dorsal and ventral edges of each HSN cell body by using ImageJ software.

For analysis of the anterior–posterior orientation of UNC-40::GFP along the dorsal surface, the dorsal segment was geometrically divided into three equal lengths (dorsal anterior, dorsal central and dorsal posterior segments). The line-scan intensity plots of each of these segments were recorded. ANOVA (http://www.physics.csbsju.edu/stats/anova.html) test was used to determine if there is a significant difference between intensities of three segments. The dorsal distribution was considered uniform if p≥0.05 and was considered asymmetrical if p≤0.05. Within an asymmetric population, the highest percent intensity was considered to localize UNC-40::GFP to either anterior, posterior or central domain of the dorsal surface. In addition, UNC-40::GFP is regarded as anterior-posteriorly localized if both anterior and posterior regions show significantly higher intensities than the central region.

### Simulations

Simulations were performed using MATLAB. Based on the probability of dorsal, ventral, anterior, and posterior outgrowth for a mutant (Table 1), a probability was assigned for the direction of each step of a random walk moving up, down, left or right, respectively. Random walks of 250 equal size steps were plotted.

**Table 1:** Axon Protrusion from the HSN Cell Body.

	direction of axon protrusion					
	dorsal	ventral	anterior	posterior	multipolar	
	%	%	%	%	%	n
wild-type	0	95±2	3±1	1±1	1±1	221
*unc-6(ev400)*	1±1	1±1	57±3	23±1	18±1	118
*unc-40(e1430)*	1±1	2±1	53±2	24±2	20±1	133
*unc-5(e53)*	0	75±2	19±2	1±1	5±1	211
*unc-52(e998)*	0	71±3	22±1	1±1	6±1	222
*unc-52(e444)*	0	60±2	28±2	8±2	4±1	96
*unc-97(su110)*	1±1	85±2	7±1	3±1	3±1	167
*unc-112(r367)*	1±1	71±2	6±1	0	22±2	177
*vab-19(e1036)*	0	77±3	15±2	1±1	7±1	191
*unc-52(e998);unc-5(e53)*	0	61±2	30±2	4±1	5±1	145
*unc-40(e1430); unc-52(e998)*	12±1	4±1	23±2	31±2	30±3	88
*unc-52(e998); unc-6(ev400)*	14±2	4±1	33±3	26±2	23±2	168
*unc-40(e1430); unc-52(e444)*	17±2	4±1	30±2	25±2	24±2	153
*unc-52(e444); unc-6(ev400)*	13±2	2±1	34±3	24±3	27±3	149
*unc-5(e53); unc-112(r367)*	0	58±3	25±1	5±1	12±1	91
*unc-40(e1430); unc-112(r367)*	12±1	4±1	27±2	24±1	33±3	139
*vab-19(e1036); unc-6(ev400)* 1	11±2	3±1	37±1	24±1	25±2	150
*unc-52(e444); vab-19(e1036)*	0	58±3	30±2	6±1	6±1	156
*mig-15(rh80)*	1±0	21±1	40±4	18±3	20±2	339
*mig-15(rh326)*	2±1	15±1	24±3	11±3	48±8	131
*gmEx593[Punc-86::MIG-15]*	0	84±3	15±3	0	1±1	68
*gmEx593;mig-15(rh326)*	0	63±1	29±9	6±5	3±3	70
*urEx386[Punc-86::mig-15(KD)]*	1±2	49±10	18±1	21±10	7±1	146
*urEx386;unc-6(ev400)*	1±1	16±1	46±4	23±3	14±1	238
*urEx386;unc-6(rh46)*	3±1	14±3	43±1	23±7	17±1	35
*ina-1(gm144)*	2±1	21±1	40±2	12±2	25±3	207
*ina-1(gm39)*	0	15±1	62±3	11±1	12±2	179
*ina-1(gm39); unc-5(e53)*	3±1	11±1	42±3	25±2	19±2	162
*ina-1(gm39); unc-6(ev400)*	15±2	2±1	40±3	21±2	22±1	175
*unc-40(e1430); ina-1(gm39)*	21±1	14±2	32±2	8±1	25±2	202

Numbers represent percentage value±SEM. Schematics depicts the direction of HSN axon protrusion.

1 Animals grown at the *vab-19(e1036)* permissive temperature of 25°C.

## Results

### Mutations that Disrupt the Muscle/epidermis Matrix Affect the Pattern of UNC-40 Asymmetric Localization in HSN

The HSN cell body sits just dorsal of a ventral longitudinal row of body-wall muscle cells, which are attached to the epidermis by structures that form a molecular link between the contractile apparatus of the muscle cell and the cuticular exoskeleton [17], [18]. These structures pass through the basement membrane that separates the muscle cells from the epidermis. Serial-section electron micrographs show that the HSN neurites invades through this basement membrane during the formation of the ventral axon [19].

Several proteins are known to play prominent roles in the structure and function of this matrix. These include: UNC-52 (perlecan), an extracellular protein of the muscle/epidermal matrix; UNC-112 (kindlin), a protein required for muscle cell-matrix adhesion complexes; VAB-19, an ankyrin repeat protein that regulates epithelial cell–matrix attachment structures; and UNC-97 (PINCH), a LIM domain adaptor protein found at extracellular matrix attachment sites. Null alleles of *unc-52* are lethal, whereas the *e444* and e998 alleles result in paralyzed, but viable animals [20]. UNC-112 is observed in the vulval, spermathecal, uterine, anal sphincter/depressor muscles, and the body wall muscles, where it colocalizes with integrin [21]. Null mutations of *unc-112* are lethal; the embryos arrest at the twofold stage and have severely disorganized body wall muscles [22]. However, animals homozygous for *r367* are viable [21]. VAB-19 is expressed in the epidermis, where it becomes localized to epidermal attachment structures including those adjacent to the body wall muscles [23]. Null mutations of *vab-19* are lethal, however the *vab-19(e1036)* allele has a cold-sensitive phenotype; at 22.5°C about 90% survive to the adult stage, whereas at 15°C most arrest at the twofold stage of embryonic development (Ding et al., 2003). The mutation disrupts epidermal elongation and muscle attachment to the epidermis. UNC-97 is expressed in touch neurons, sex muscles, and body wall muscles, where it colocalizes with integrin PAT-2/PAT-3 [24], [25]. Loss of *unc-97* function is embryonic lethal, whereas the *unc-97(su110)* allele is viable; it causes defective muscle attachments [24].

**Figure 2:**
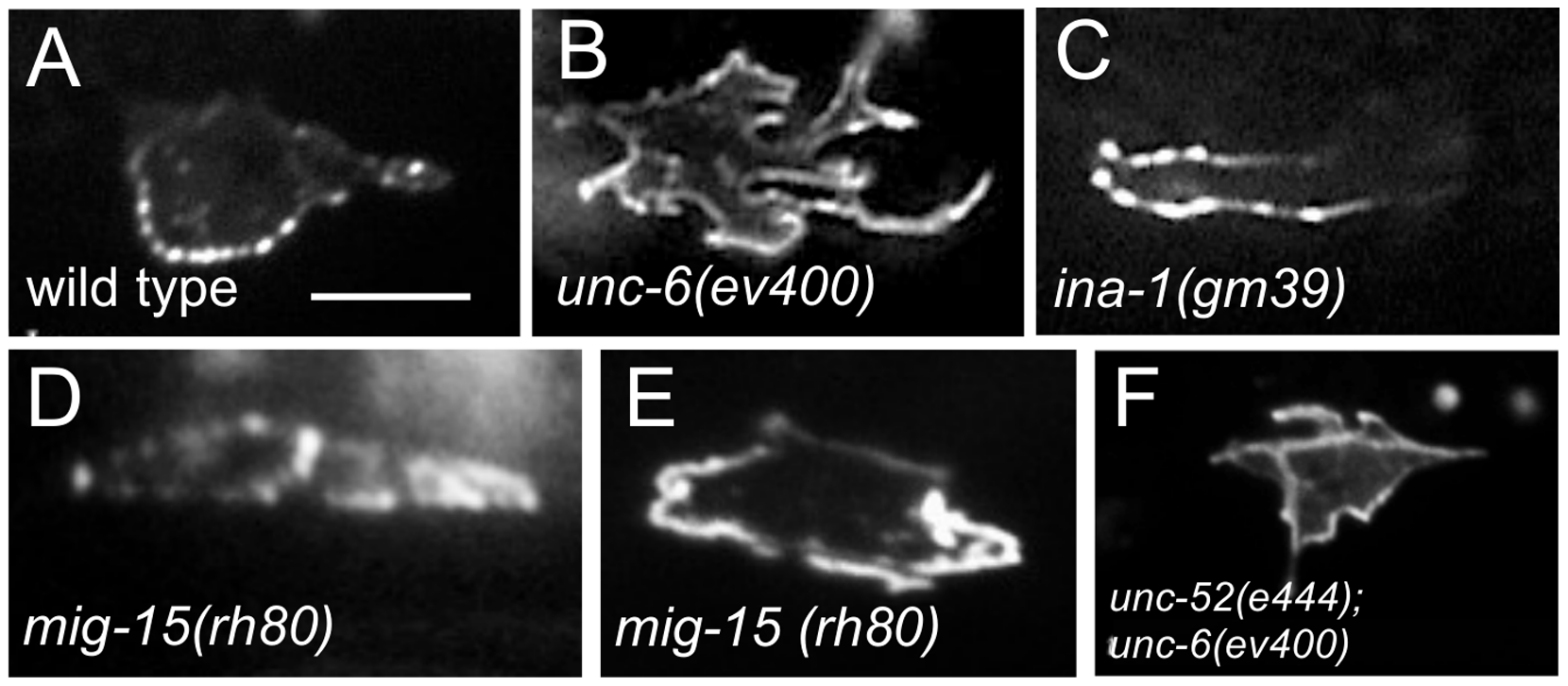
Mutations Affect Intracellular UNC-40::GFP Localization. (A–I) Photomicrographs showing examples of UNC-40::GFP localization in the HSN neuron of L2 stage larvae. Ventral is down and anterior is to the left. Scale bar: 5 µm. UNC-40::GFP is ventrally localized in the wild-type animals (A), but in *unc-6(ev400)* mutants UNC-40::GFP is evenly distributed and the cell shape is generally more irregular (B). In the mutants, UNC-40::GFP can be asymmetrically localized in the anterior (C), posterior (D) or in the anterior and posterior (E) direction. (F) Double mutants with *unc-6(ev400)* show the *unc-6(ev400)* phenotype with evenly distributed UNC-40::GFP and the more irregular cell shape.

**Figure 3:**
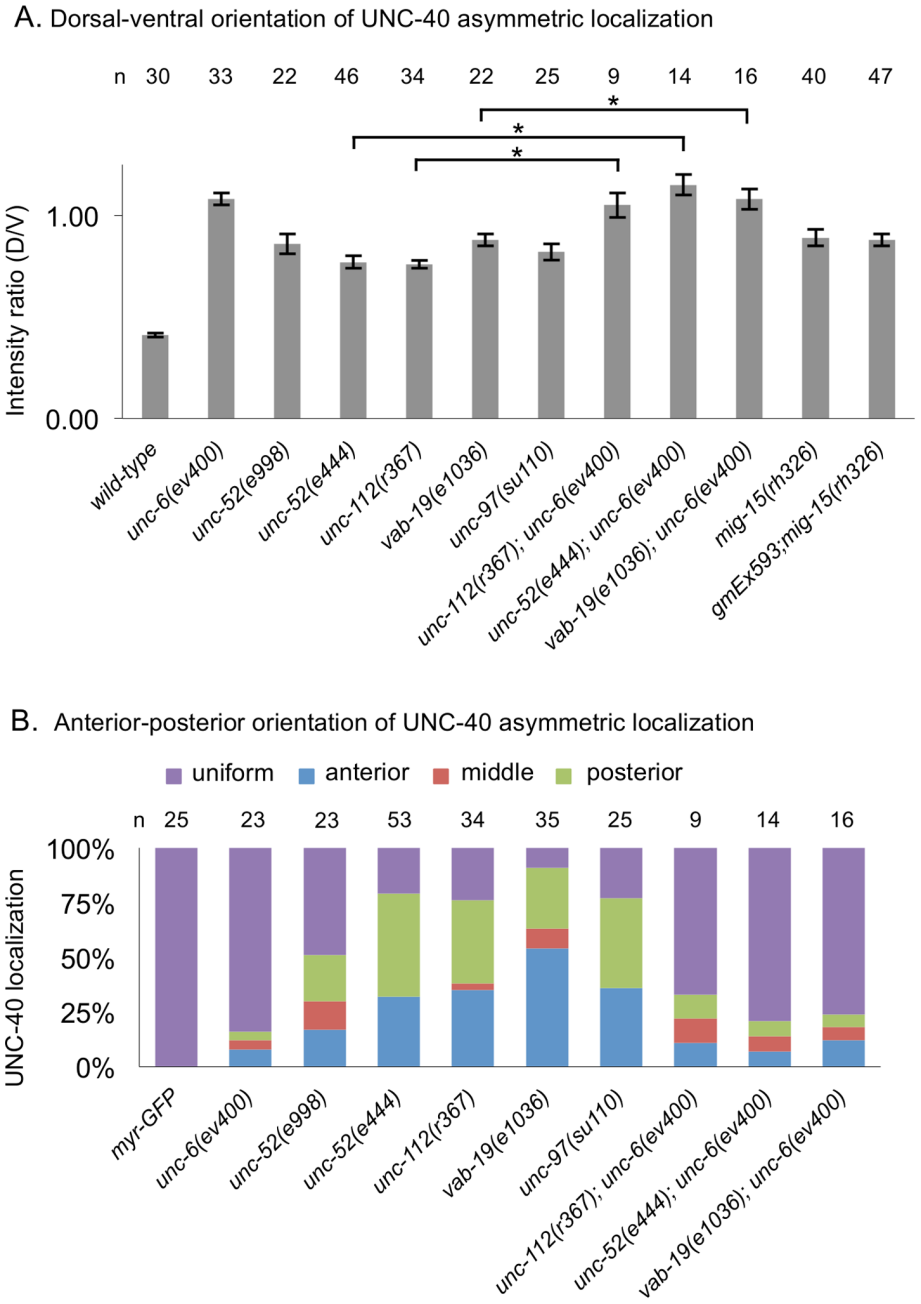
The Muscle/epidermis Extracellular Matrix Inhibits Anterior and Posterior UNC-40 Localization in Response to UNC-6. (A) Graph indicating the dorsal-ventral orientation of UNC-40::GFP in HSN. The graph shows the average ratio of dorsal-to-ventral intensity from linescan intensity plots of the UNC-40::GFP signal around the periphery of the HSN cell. UNC-40::GFP is ventrally localized in wild-type, but the ratio is different in *unc-6(−)* and the mutants. (*) statistic difference (P<0.05, one-tailed Student’s *t-*test). Error bars represent standard error of mean. (B) Graph indicating the anterior-posterior orientation of UNC-40::GFP. To determine orientation, line-scan intensity plots of the UNC-40::GFP signal across the dorsal periphery of the HSN cell were taken, the dorsal surface was geometrically divided into three equal segments, and the total intensity of each was recorded. The percent intensity was calculated for each segment and ANOVA was used to determine if there is a significant difference between the three segments (see Experimental Procedures). The measurements were taken using only the dorsal periphery in order to minimize cell shape differences. In the mutants there is a bias for anterior or posterior localization, whereas there is a uniform distribution in *unc-6(−)* mutants and in double mutants with *unc-6(−)*.

We find that the *unc-52(e998), unc-52(e444), unc-97(su110), unc-112(r367),* and *vab-19(e1036)* mutations alter the pattern of UNC-40 asymmetric localization in HSN. In particular, the mutations increase the probability of UNC-40 asymmetrically localizing in the anterior and posterior direction, while decreasing the probability of UNC-40 asymmetrically localizing in the ventral direction (Figures 2 and 3). In these experiments we first assay for the distribution of UNC-40::GFP along the dorsal-ventral axis (Figure 3A). We then assay for bias along the anterior-posterior axis by determining the UNC-40::GFP distribution along dorsal side of the neuron (Figure 3B). Whereas in wildype animals UNC-40::GFP is frequently localized ventrally and in *unc-6* mutants UNC-40::GFP is uniformly dispersed around the periphery of HSN, in these mutants there is an increase in the probability that UNC-40::GFP will localize at either the anterior or posterior sides of the neuron.

Despite any changes to the extracellular matrix caused by the mutations, HSN still responds to UNC-6. This is evident because UNC-6 signaling induces the UNC-40 asymmetric localization and in these mutants UNC-40 becomes asymmetrically localized. In fact, double mutants, *unc-52(e444); unc-6(ev400), unc-112(r367); unc-6(ev400),* and *vab-19(e1036); unc-6(ev400),* are similar to *unc-6(ev400)* (Figure 3A, B), *i.e.* there is a more uniform distribution of UNC-40::GFP around the periphery of HSN (Figure 2F). Therefore the effect that the *unc-52(e998), unc-52(e444), unc-112(r367),* and *vab-19(e1036)* mutations have on the pattern of intracellular UNC-40 asymmetric localization depends on UNC-6 signaling.

**Figure 4:**
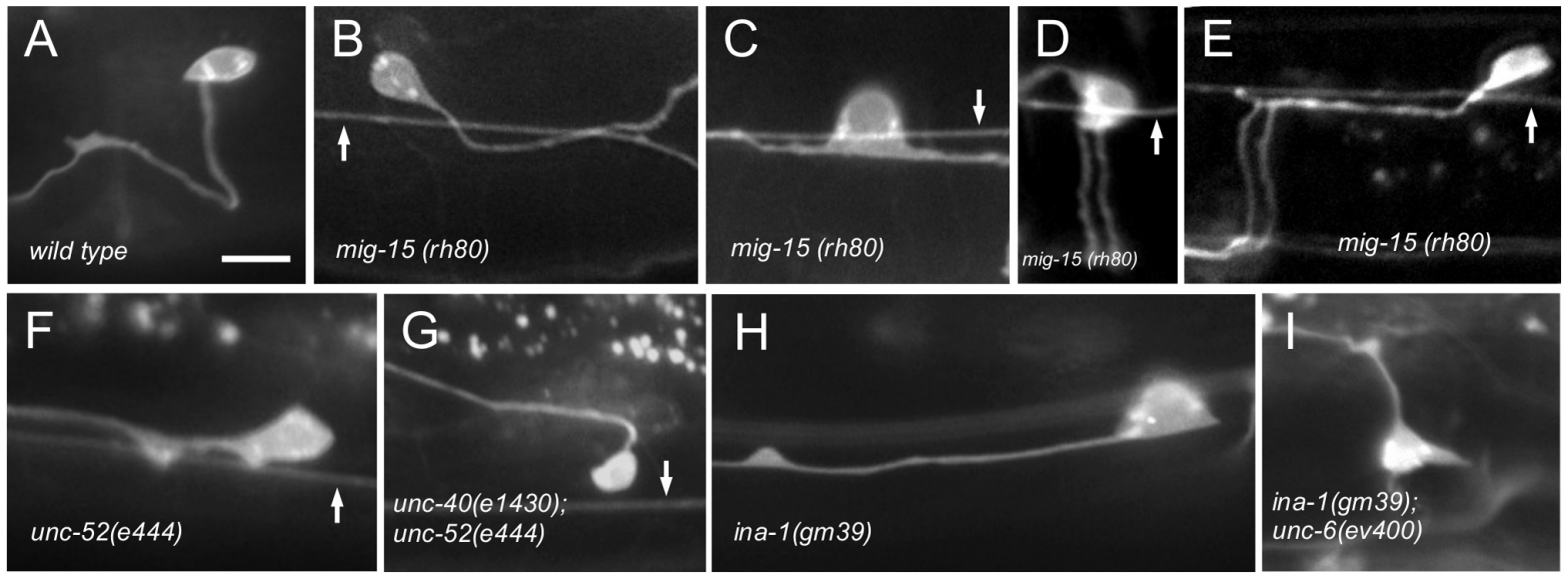
Mutations Affect the Direction of HSN Axon Protrusion. (A–G) Photomicrographs of L4 stage animals showing examples of the protrusion of the axon from the HSN cell body in wild-type and mutant animals. Ventral is down and anterior is to the left. Arrow indicates the PLM axon, if in the focal plane. Scale bar: 10 µm. (A) In wild-type animals, the HSN axon protrudes ventrally from the cell body. After reaching the ventral nerve chord the axon extends anteriorly and defasciculates from the cord to form synapses at the vulva. (B–E) In *mig-15* mutants, the HSN axon can protrude posterior (B), anterior and posterior (C), ventral (D) or anterior (E). Most axons in *mig-15* mutants that anteriorly or posteriorly protrude from the cell body eventually turn ventral. Only a few fail to reach the ventral nerve core (8% ±2, n = 100). (D–E) As axons cross the muscle/epidermis interface (see Figure 1B) they sometimes split into two parallel processes (26% ±1, n = 100). (F–I) As in *mig-15* mutants, the axon protrudes anteriorly, posteriorly, or ventrally in *unc-52*, *ina-1*, *unc-97*, *unc-112,* and *vab-19* mutants. In double mutants with either *unc-40(e1430)* (G) or *unc-6(ev400)* (I) the axon will sometimes protrude in the dorsal direction.

We find that these mutations also affect the direction in which the axon protrudes from the HSN cell body (Table 1). In particular, the percent of neurons that have an axon protruding ventrally decreases, whereas the percent that have an axon protruding anteriorly or posteriorly increases. We also tested double mutations; *unc-40(e1430); unc-52(e998), unc-52(e998); unc-6(ev400), unc-40(e1430); unc-52(e444), unc-52(e444); unc-6(ev400), unc-40(e1430); unc-112(r367),* and *vab-19(e1036); unc-6(ev400).* In these cases, the probability of dorsally directed protrusion is greater than in any of the single mutants (Figure 4F, Table 1).

We previously found that the UNC-6 receptor UNC-5 also can play a role in the UNC-6 guidance of the HSN axon [6]. However, we find that the directional pattern of axon protrusion in *unc-52(e998);unc-5(e53)* and *unc-5(e53); unc-112(r367)* mutants is similar to the pattern observed in *unc-52(e998)* and *unc-112(r367)* mutants, respectively (Table 1). This suggests that the effects caused by the *unc-52(e998)* and *unc-112(r367)* mutations on the direction of protrusion do not require UNC-5 function. This is consistent with the previous finding that UNC-5 functions through a parallel pathway to induce UNC-40 asymmetric localization in HSN [6].

### *mig-15* and *ina-1* Mutations Increase the Probability of Anteriorly and Posteriorly Directed UNC-40 Axon Outgrowth Activity

We hypothesize that integrin signaling in HSN may regulate the response to the muscle/epidermal matrix. Integrins are heterodimeric transmembrane receptors and are one of the major cell surface receptors used to mediate cell-matrix interactions [26]. Integrin is required to localize UNC-40 to the invasive membrane of the anchor cell, which extends through an extracellular matrix [11], [13]. *C. elegans* has one beta (PAT-3) integrin chain and two alpha (INA-1 and PAT-2) integrin chains. INA-1 is expressed in migrating neurons, the pharynx, and the DTCs, but has not been detected in the body-wall muscles [27], [28]. It’s been reported that the integrin INA-1/PAT-3 interacts with MIG-15, which can act cell-autonomously to regulate UNC-6 axon guidance [16]. Genetic evidence would support the hypothesis that INA-1/MIG-15 signaling regulates the HSN response to the muscle/epidermal matrix if *ina-1* and *mig-15* mutations cause the same intracellular UNC-40 localization phenotypes as the mutations that perturb the muscle/epidermal matrix.

**Figure 5:**
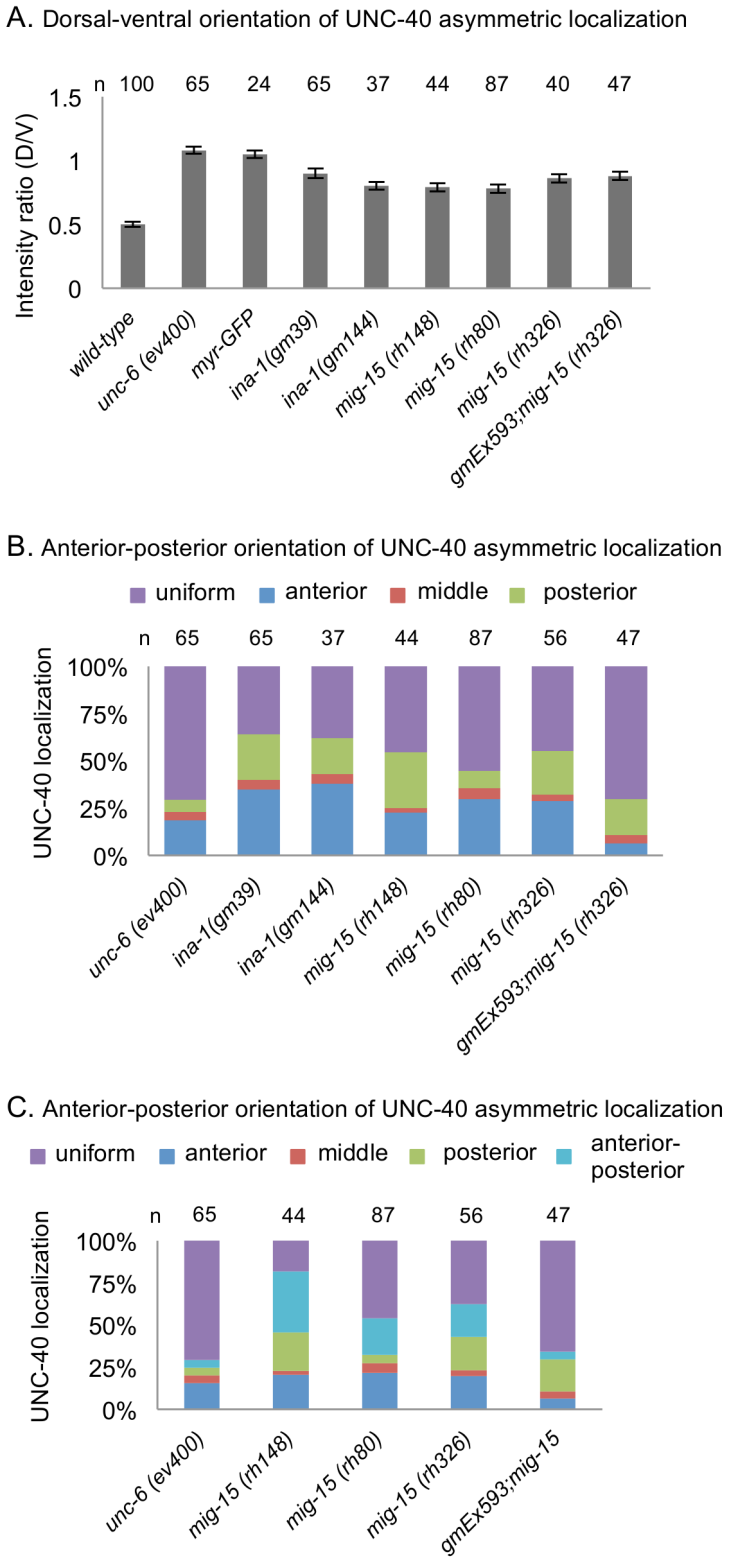
Integrin Signaling Inhibits Anterior and Posterior UNC-40 Localization. (A) Graph indicating the dorsal-ventral orientation of UNC-40::GFP in HSN. The graph shows the average ratio of dorsal-to-ventral intensity from linescan intensity plots of the UNC-40::GFP signal around the periphery of the HSN cell. Wild-type animals show a strong ventral bias, whereas there is a uniform distribution in *unc-6(−)* mutants. In *ina-1* and *mig-15* mutants there is an intermediate phenotype indicating a weak bias for ventral localization, as well as enrichment for localization at other sites compared to wild-type. (B) Graph indicating the anterior-posterior orientation of UNC-40::GFP. To determine orientation, line-scan intensity plots of the UNC-40::GFP signal across the dorsal periphery of the HSN cell were taken and analyzed as in Figure 3B. In the *ina-1* and *mig-15* mutants there is a bias for anterior or posterior localization. (C) Graph indicating the anterior-posterior orientation of UNC-40::GFP, including cases where there is significant localization at both the anterior and posterior sides of the neuron. Orientation was determined as in B. However, if the anterior and posterior were not significantly different and was greater than the middle segment it was scored as anterior-posterior (see Material and Methods). In the mutants there is a bias for localization in both the anterior and posterior direction. Error bars represent standard error of mean. n >15.

We find that *mig-15* and *ina-1* mutations similarly alter the pattern of UNC-40 asymmetric localization in HSN. That is, the mutations decrease the probability of UNC-40 asymmetrically localizing ventrally, while increasing the probability of UNC-40 asymmetrically localizing in the anterior and posterior direction (Figure 5). We further observe that in *ina-1* and *mig-15* mutants the direction to which the HSN axon protrudes similarly varies (Figure 1C, Figure 4). In the mutants there is a higher probability that the axon will protrude in the anterior or posterior directions (Table 1). The *mig-15* mutations, *rh326* and *rh80,* cause nonsense changes of Q439STOP and W898STOP, respectively; *rh326* may be a null allele, whereas *rh80* is thought to retain some *mig-15* activity [14]. Null mutations in *ina-1* are early larval lethal [28]. The *gm39* mutation is a missense mutation within the putative ligand binding β propeller region, whereas *gm144* is a missense mutation adjacent to the transmembrane domain [28].

We observed that in *mig-15* mutants the HSN neuron will often become bipolar, sending an axon in both the anterior or posterior directions. To explore further the association of this phenotype with the intracellular localization of UNC-40 we rescored the localization of UNC-40::GFP in HSN taking into consideration cases where the intensity at both the anterior and posterior sides where nearly equal and both were greater than the central domain (Figure 2E). Consistent with the frequent multipolar morphology of HSN in *mig-15* mutants (Figure 4C), the probability that UNC-40::GFP will localize primarily to both the anterior and posterior sides of the neuron is higher than in wild-type animals (Figure 5C).

### *mig-15* Functions Cell Autonomously to Regulate the Pattern of UNC-40 Asymmetric Localization in HSN

To test whether *mig-15* functions within HSN, we performed cell-specific RNAi-mediated knockdown of the *mig-15* gene using the *unc-86* promoter, which is expressed in HSN and a limited number of cells, but not in the body-wall muscles [29]. Animals with a wild-type background subjected to RNAi exhibited a HSN axon protrusion phenotype similar to the *mig-15* mutants (Table 1). We also confirmed the previous report [14] that transgene expression of *mig-15* under the *unc-86* promoter can partially rescue the *mig-15* axon protrusion phenotype (Table 1). Further, we found that expression of this transgene also partially rescues the intracellular UNC-40::GFP localization pattern. In the transgenic strain, *gmEx593;mig-15(rh326),* the probability of anterior or posterior localization is reduced in comparison to *mig-15(rh326)* mutant (Figure 5B, C). A significant difference in the distribution of UNC-40::GFP along the dorsal-ventral axis is not observed, likely because the transgene only partially rescues the phenotypes (Figure 5A). Similar experiments to test the cell-autonomous function of *ina-1* in HSN were problematic. In fact, we found that the *ina-1* mutations are lethal in combination with the *unc-6(ev400)* mutation and the UNC-40::GFP transgene. This may reflect a critical relationship between integrin and netrin signaling.

### Mutations Delay Axon Formation

Previously it was noted that *unc-6* and *unc-40* mutations can alter the timing of HSN axon development [19]. In comparison to wild-type animals, in *unc-6* mutants UNC-40 remains more uniformly distributed across HSN and the initial neurites that form do not develop a strong bias for the ventral direction. Normally by the early L2 larval stage HSN is polarized ventrally with neurites primarily restricted to the ventral side where a leading edge forms. Around the L3–L4 transition a single ventral axon becomes evident. However in *unc-6* and *unc-40* mutants axon development is delayed and neurite extension is not confined to the ventral side during the L3 stage. A predominate axon, which protrudes anteriorly, eventually forms however not until the L4 stage [19]. In Wnt/PCP mutants, which have *egl-20*, *mig-1*, or *vang-1* mutations, the axon forms ventrally in response to UNC-6 but the development is delayed [6]. In these mutants, neurites are not clearly ventrally oriented until around the L3–L4 transition. We hypothesize that this phenotype is the result of the direction of UNC-40 outgrowth activity randomly fluctuating in response to UNC-6. This fluctuation occurs because the Wnt signaling normally inhibit anteriorly and posteriorly directed UNC-40-mediated axon outgrowth and in the mutants the probability of UNC-40 localization at the anterior and posterior sides increases, whereas the probability of localization at the ventral side decreases [6].

**Figure 6:**
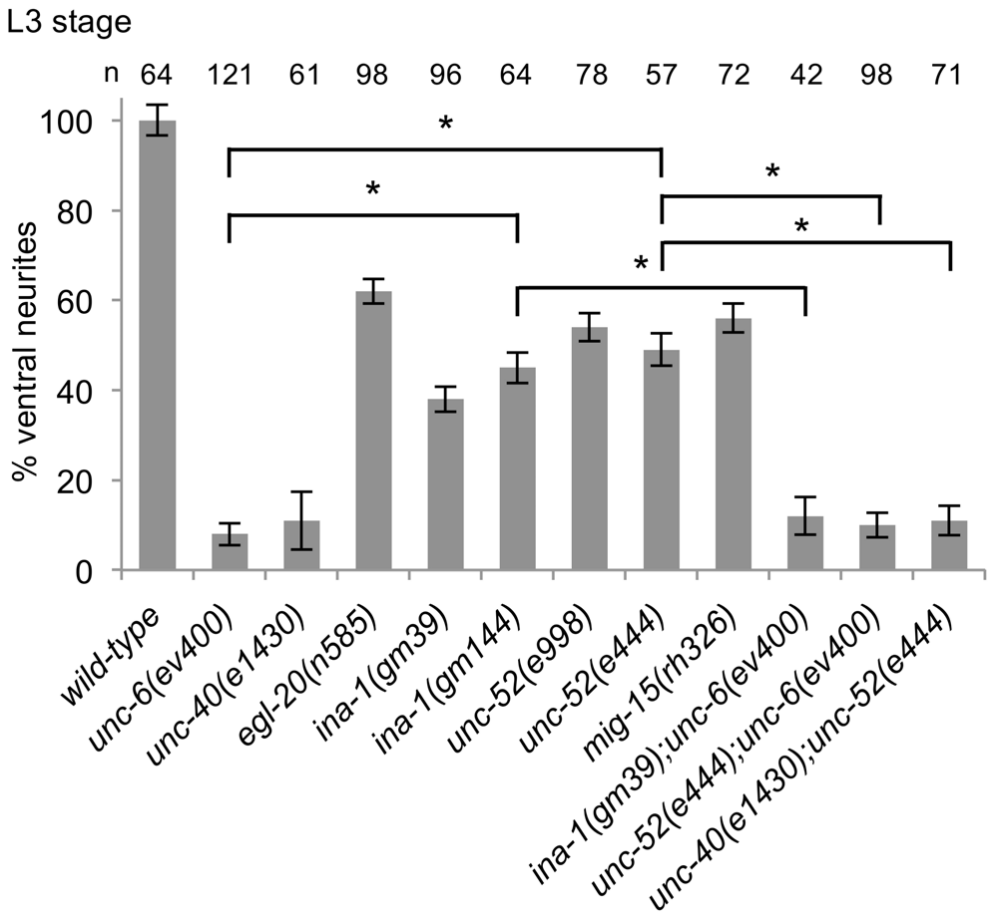
Mutations delay the development of predominantly ventral neurites. (A) The percentage of HSN neurons with predominantly ventral neurites in mid-L3. Whereas in wild-type animals there are predominately ventral neurites by this stage, in the single mutants there is a delay. In all strains an axon will form in the L4 stage. Double mutants suggest that the development of predominate ventral neurites at the L3 stage in *ina-1* and *unc-52* mutants depends on UNC-6 signaling. Error bars indicated the SEM; n values are indicated above each column. Significant differences (two-tailed *t*-test), *P<0.001.

We examined the duration of axon development in *ina-1(gm39)*, *ina-1(gm144)*, *unc-52(e998)*, *unc-52(e444)*, and *mig-15(rh326)* mutants. Similar to the Wnt/PCP mutants, we find that the neurons don’t clearly show ventrally oriented neurites until around the L3–L4 transition (Figure 6). Further, the phenotype of the double mutants, *ina-1(gm39);unc-6(ev400)*, *unc-52(e444);unc-6(ev400)* and *unc-40(e1430);unc-52(e444)*, is similar to the *unc-6(ev400)* or *unc-40(e1430)* mutants. These results are consistent with the idea that INA-1, UNC-52 and MIG-15 function to promote the UNC-6 and UNC-40 activity responsible for directing axon formation in the ventral direction.

### Mutations Cause Precocious Axon Formation

**Figure 7:**
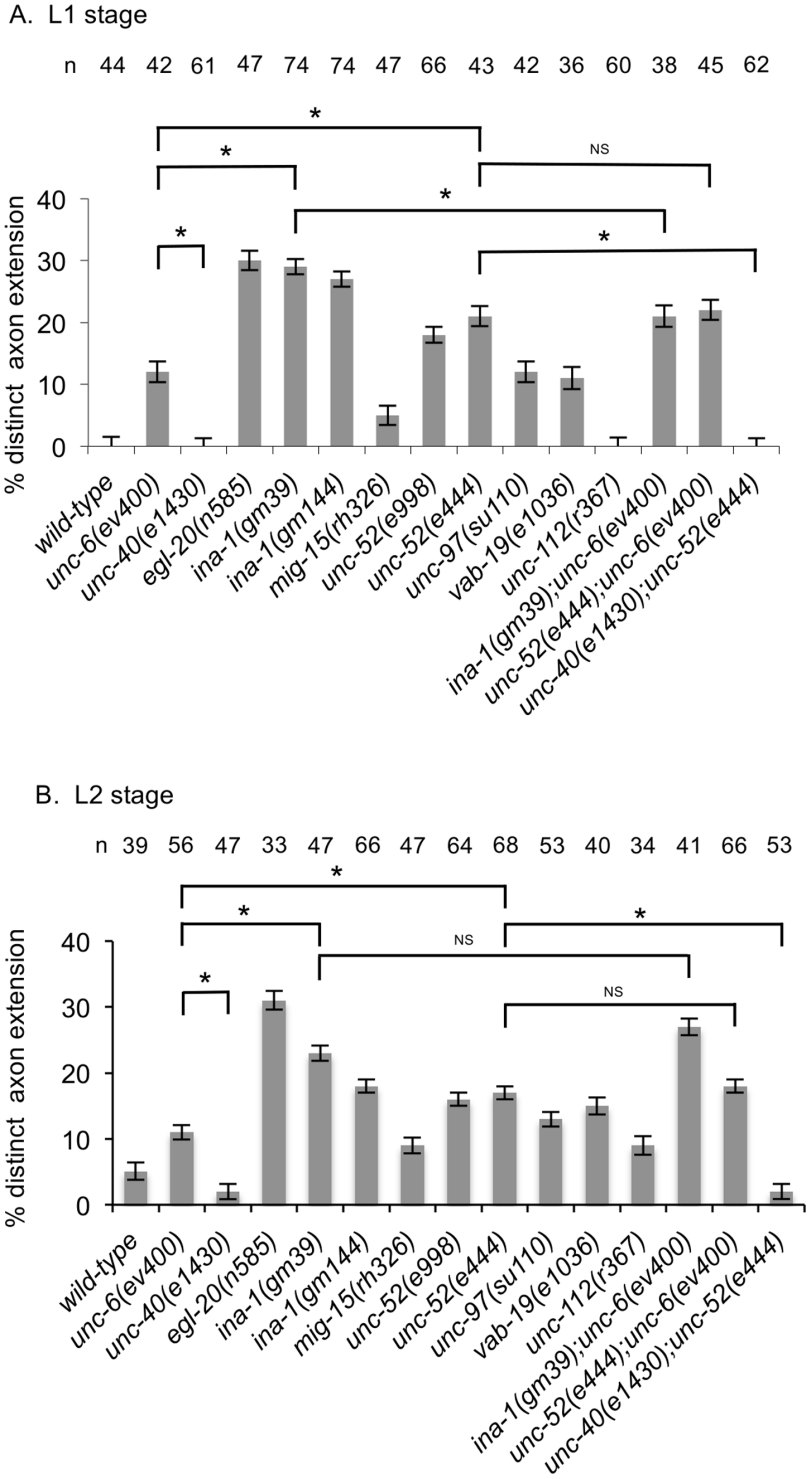
Mutations cause the development of precocious axons. (A) Percentage of HSN neurons with a distinct single axon in the L1 stage. A neuron was scored as having a distinct axon extension if the length of the protrusion was at least twice the anterior to posterior length of the cell body. (B) Percentage of HSN neurons with a distinct single axon in the L2 stage. Whereas in wild-type animals a distinct axon does not form until the early L4 stage, in the mutants axons are observed at earlier stages. The precocious effect of the *unc-52(e444)* mutation requires UNC-40 function, but not UNC-6. Error bars indicated the SEM; n values are indicated above each column. Significant differences (two-tailed *t*-test), *P<0.001; NS P = 0.6.

Whereas only immature neurites are observed at the L1 and L2 stage in wild-type animals, some mutations can cause early development of a distinct axon, which is defined as a process that extends greater than a distance equal to two times the anterior to posterior length of the cell body. This phenotype has been observed in mutants for EGL-20, MIG-1, and VANG-1 [6] and in mutants for the *lin-4* microRNA and the POU homeodomain transcription factor UNC-86 [30], [31]. We now report that the *ina-1(gm39), ina-1(gm144), unc-52(e998),* and *unc-52(e444)* mutations also cause this precocious phenotype. Distinct axon extensions can be observed in the L1 stage (Figure 7A) and the L2 stage (Figure 7B). The phenotype of the double mutant, *unc-52(e444);unc-6(ev400),* is similar to that of the single *unc-52(e444)* mutant. This suggests that the precocious development caused by the loss of UNC-52 function is independent of UNC-6 signaling. However, we also observe that the precocious phenotype is suppressed in *unc-40(e1430);unc-52(e444)* double mutants, suggesting that the precocious axon in *unc-52(e444)* mutants is caused by UNC-40 activity that is independent of UNC-6.

### The Direction of AVM Axon Protrusion is Less Affected by the Mutations

The AVM axon also migrates ventrally through the muscle/epidermal matrix and the guidance of AVM also requires UNC-6 and UNC-40. However, AVM is a mechanosensory neuron that develops a specialized extracellular matrix and forms attachments with the epidermis; in fact, the epidermis envelops the AVM process [32]. We examined the direction of the axon protrusion from the AVM neuron and found that unlike with HSN, the *unc-52(e998)*, *ina-1(gm39)*, *mig-15(rh80)*, *unc-97(su110)*, and *vab-19(e1036)* mutations have little affect on the direction of axon protrusion (Table 2). Further, we did not notice any significant differences in the rate of AVM axon development between wild-type animals and *unc-6(ev400)*, *unc-40(e1430)*, *egl-20(n585)*, *ina-1(gm39)*, *unc-52(e444)*, or *mig-15(rh326)* mutants (data not shown).

**Table 2:** Axon Protrusion from the AVM Cell Body.

	direction of axon protrusion					
	dorsal	ventral	anterior	posterior	multipolar	
	%	%	%	%	%	n
wild-type	0	100	0	0	0	121
*unc-6(ev400)*	0	62±2	38±1	0	0	226
*unc-52(e998)*	0	99±1	0	0	1±1	110
*ina-1(gm39)*	0	92±2	2±1	5±1	1±1	121
*mig-15(rh80)*	0	95±2	4±1	1±1	0	76
*unc-97(su110)*	0	93±2	5±1	2±1	0	135
*vab-19(e1036)*	0	100	0	0	0	101

Numbers represent percentage value±SEM. Schematics depicts the direction of AVM axon protrusion.

## Discussion

We previously showed that the directional response of a neuron to UNC-6 is stochastic; *i.e.,* there is a probability of UNC-40-mediated axon outgrowth activity occurring in any direction [6], [7]. We have proposed that the direction of UNC-6 guidance is determined by asymmetric cues that create a bias which directs UNC-40-mediated axon outgrowth on average, over time, towards one side of the neuron [6]. In this paper we provide evidence that the extracellular matrix acts as such a cue and provides directional information for UNC-6 guidance.

### The Extracellular Matrix Provides Directional Information for UNC-6 Guidance

Axons respond to the surrounding environment and follow highly stereotyped and reproducible trajectories to their targets [33]. During the past few decades many molecular signaling molecules that promote and guide axon outgrowth have been identified. Netrins are commonly described as tropic cues that are bifunctional, they act both as chemoattractants and chemorepellants [34]–[36]. A chemoattractant stimulates axon protrusion towards the site of ligand-receptor binding [37]. In this model, the attractive UNC-6 response in HSN is greater towards the ventral source of UNC-6 because of the UNC-6 gradient and, therefore, the axon protrudes ventrally towards the UNC-6 source. In contrast, the stochastic model predicts that UNC-6 induces a stochastic fluctuation in the direction of UNC-40-mediated axon outgrowth. Since this response is non-directional, UNC-6 does not intrinsically function as either an attractant or repellent.

The results presented here indicate that the muscle/epidermis extracellular matrix is a directional cue for UNC-6 guidance. Matrix molecules, such as UNC-52 perlecan, may not themselves cause a directional response from the neuron. However, when UNC-6 induces the direction of UNC-40-mediated axon outgrowth activity to stochastically fluctuate within a neuron the interaction between the matrix and the neuron can cause directionality by increasing the probability that UNC-40-mediated axon outgrowth occurs at the surface of the neuron that is in contact with the matrix. We show that when the muscle/epidermis extracellular matrix is disrupted by different mutations, UNC-6 still induces the asymmetric localization of UNC-40 in HSN, but the probability of anterior and posterior UNC-40 localization and axon outgrowth increases, whereas the probability of ventrally directed localization and outgrowth decreases. Consistent with inducing fluctuations in the direction of UNC-40-mediated axon outgrowth activity and with movement described by a biased random walk model, the HSN axon is guided by UNC-6 to the ventral nerve cord in the mutants even though axon outgrowth from the HSN cell body can be directed anteriorly or posteriorly.

**Figure 8:**
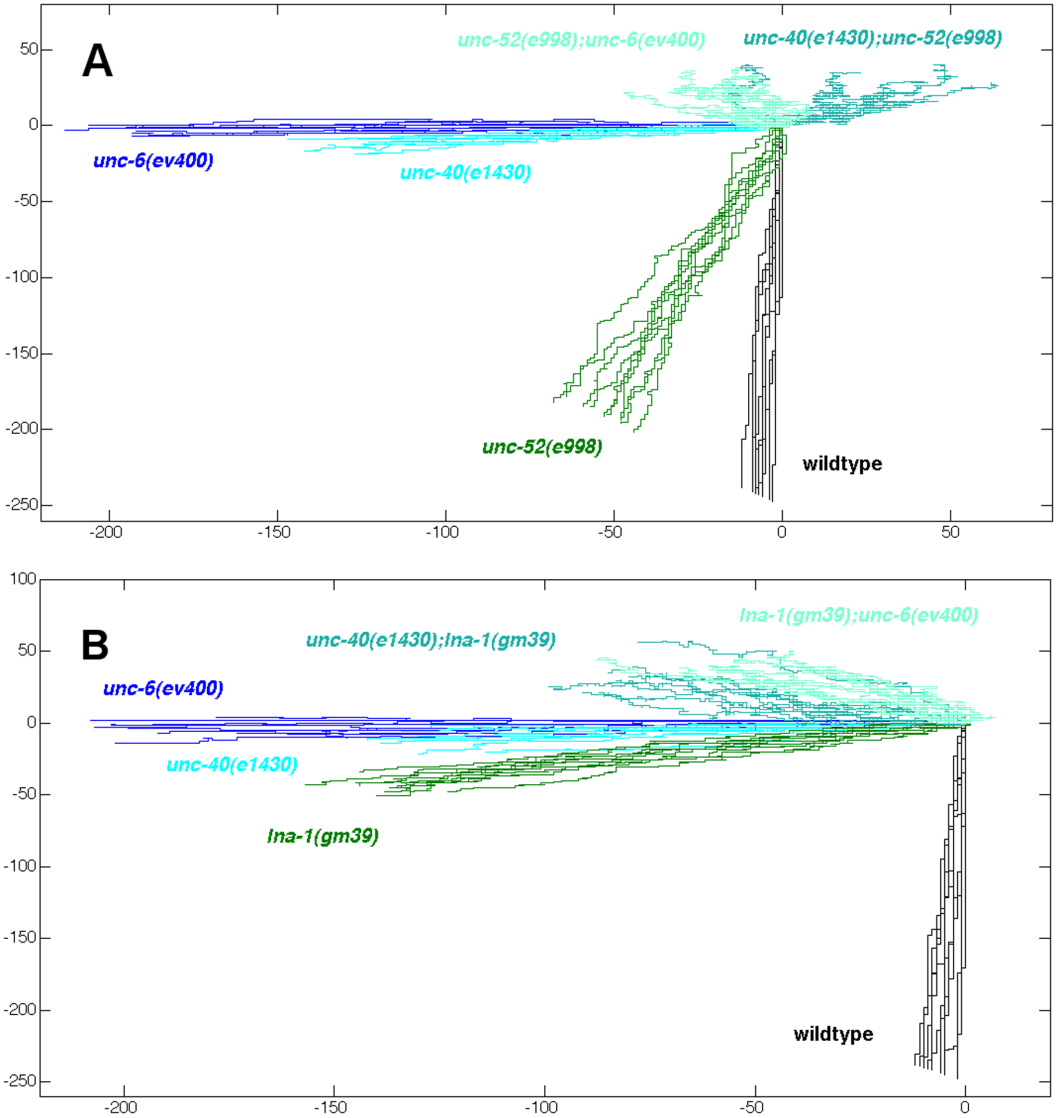
The directional bias during the initial protrusion of the HSN axon is altered by the mutations. Simulated random walks of 250 steps were plotted from an origin (0, 0). The walks were generated using the probabilities of outgrowth in the dorsal, ventral, anterior, and posterior direction, as listed for each mutant in Table 1. See text for details. (A) Plots generated to compare the effect that UNC-52 (perlecan) has on the directional bias. In the *unc-52(e998)* mutant there is a bias for ventral outgrowth. This bias is eliminated in *unc-52(e998); unc-6(ev400)* and *unc-40(e1430);unc-52(e998)* mutants. The difference suggests that UNC-52 alters the direction bias of UNC-6 axon guidance by altering the probability of UNC-40-mediated axon outgrowth in each direction. This is consistent with the evidence that *unc-52(e998)* alters the probability of UNC-40-mediated axon outgrowth activity in each direction (Figure 3). The direction of the bias in the double mutants is different than that in *unc-6(ev400)* and *unc-40(e1430)* mutants. There is a strong bias for axon outgrowth in the anterior direction in *unc-6(ev400)* and *unc-40(e1430)* mutants. This outgrowth is UNC-40-independent, suggesting that the *unc-52(e998)* mutation also effects UNC-40-independent outgrowth activity in the double mutants. (B) Plots generated to compare the effect that INA-1 (integrin) has on the directional bias. In the *ina-1(gm39)* mutant there is a bias for ventral outgrowth. This ventral bias is eliminated in *ina-1(gm39); unc-6(ev400)* and *unc-40(e1430); ina-1(gm39)* mutants. The difference suggests that INA-1 alters the direction bias of UNC-6 axon guidance by altering the probability of UNC-40-mediated axon outgrowth in each direction. This is consistent with the evidence that *ina-1(gm39)* alters the probability of UNC-40-mediated axon outgrowth activity in each direction (Figure 5). The direction of the bias in the double mutants is different than that in *unc-6(ev400)* and *unc-40(e1430)* mutants. There is a strong bias for axon outgrowth in the anterior direction in *unc-6(ev400)* and *unc-40(e1430)* mutants. Because the anterior outgrowth is UNC-40-independent, the result suggests that the *ina-1(gm39)* mutation must also effect UNC-40-independent outgrowth activity in the double mutants.

Figure 8 graphically illustrates how altering the probability of outgrowth in each direction can influence the direction of guidance. The probability that an axon will protrude in a specific direction is controlled in part by the fluctuations of UNC-40 axon outgrowth activity that occur as the leading edge forms. These fluctuations are described in Figures 3 and 5. Whereas the axon protrudes from the ventral side of the cell body in wild-type animals, in *unc-52* and *ina-1* mutants there is a probability that the axon will also protrude from other sides (Table 1). This is consistent with the observations that the probability of UNC-40 localizing to each side of the neuron is altered by the mutations (Figures 3 and 5). To visualize how this fluctuation effect a directional bias, we simulated a random walk of 250 steps (arbitrarily chosen) based on the probability of axon outgrowth in the dorsal, ventral, anterior, and posterior directions (for simplicity the multipolar phenotype was ignored). The probabilities are given in Table 1. For each mutant, 10 simulations are plotted. The results graphically compare the directional bias of the system at the time of early axon protrusion and they depict how each mutation produces a different directional bias by altering the probabilities of outgrowth in each direction.

Figure 8 shows that at the time of early axon protrusion there is a bias for axon outgrowth in the ventral direction in *unc-52* and *ina-1* mutants. This ventral bias depends on UNC-40 and UNC-6, since in the double mutants there is no ventral bias. It is worth noting that in mutants, such as *unc-40(e1430);ina-1(gm39),* a substantial number of axons will initially protrude in the ventral direction. Nevertheless, the system does not create a ventral directional bias. The graphs also illustrate the strong bias for anteriorly directed axon outgrowth in *unc-6* and *unc-40* mutants and that the *unc-52* and *ina-1*mutations also affect this UNC-40-independent mediated activity.

If a mutation did not create any fluctuations in the direction of axon outgrowth activity, *i.e.* there was always a 100% probability of outgrowth in just one direction, than a corresponding line in Figure 8 would extend as a straight-line of 250 steps from the origin. However, a property of an object undergoing a random walk is that the distance increases only in proportion to the square root of the number of steps. We suggest that fluctuations in the direction of axon outgrowth activity could underlie the slower formation of the axon in the mutant (Figure 6). Figure 8 illustrates that mutations that cause fluctuations produce lines where the average distance from the origin to the endpoint is shorter than wildtype.

The ability of the direction of axon outgrowth activity to fluctuate could also underlie precocious axon formation in mutants (Figure 7). Before leading edge formation, the HSN neuron dynamically extends short neurites in different directions before they become restricted to the ventral side of the neuron [19]. In the mutants, there is an increase in the fluctuation of the direction of axon outgrowth activity. This could increase the probability that the early protrusions do not effectively retract and could increase the probability that an early protrusion continues to extend.

What does our analysis reveal about the molecular mechanisms of axon outgrowth? We observe that the *unc-52* and *ina-1* mutations alter the probability of UNC-40 localizing to each side of neuron (Figures 3 and 5). This suggests that the probabilities are influenced by integrin-mediated interactions between the cell surface of the neuron and the muscle/epidermis extracellular matrix. This contact may regulate the rate of assembly and disassembly of UNC-40 complexes at the surface membrane of the neuron. Studies in vertebrates suggest that integrins can act as receptors for Netrin-1 [38], [39] and so it is possible that INA-1 directly potentiates UNC-40 complex formation in response to UNC-6 that is found within the muscle/epidermis matrix. Other studies report that the enrichment of UNC-40 at the site of protrusion is important and that INA-1 likely regulates the stabilization or trafficking of UNC-40 at the cell membrane in contact with the matrix [11], [13]. The stochastic model only requires that somehow the interactions alter the probabilities of UNC-40 axon outgrowth activity occurring at each side of the neuron. The interactions do not necessarily have to affect axon outgrowth *pre se.* For example, contact with the matrix might alter some property of the neuron’s membrane that in turn leads to an event that influences the probability of UNC-40 complexes assembling or disassembling at that site.

The probabilistic approach used to generate Figure 8 does not address the molecular mechanisms of axon outgrowth. It is based on the idea that the direction of axon outgrowth activity randomly fluctuates in response to the extracellular guidance cues [6], [7]. We use probabilities to describe the mass effect that the molecular mechanisms have on the macroscopic movement of the axon. Although this approach does not describe molecular mechanisms of axon outgrowth, it may prove extremely valuable for understanding the effect that a mutation has on determining the direction of guidance. This approach differs from a deterministic approach. Currently a deterministic model considers that the axon outgrowth response to a guidance cue is directional (attractive or repulsive) [40]. There is no randomness involved; the direction of outgrowth determines the direction of guidance. Therefore, understanding the molecular mechanisms by which guidance cues cause an attractive or repulsive outgrowth also explains how the direction of axon guidance is determined. However, if the direction of axon outgrowth activity stochastically fluctuates in response to a guidance cue then the direction of axon outgrowth does not reveal the direction of guidance. This point is illustrated by Table 1 and Figure 8. The initial axon outgrowth in a mutant (Table 1) is often not in the direction of guidance (Figure 8). The probabilistic approach predicts that the direction of guidance is determined by the succession of randomly directed outgrowth. Based on this concept the effect a mutation has on determining the direction of axon guidance is described by how it affects the probabilities of outgrowth in each direction.

### A General Model for Netrin/DCC Activity

Besides guiding axons, UNC-6 and UNC-40 signaling in *C. elegans* plays a role in anchor cell invasion and in the formation of presynaptic specializations [12], [13], [41]–[43]. In other organisms, netrins play many diverse roles, including regulating vasculature, lung, pancreas, muscle and mammary gland development [44]. Netrins and their receptors also have been implicated in the regulation of tumor development and progression [45], [46].

The model we propose could have broad implications. In general netrin/DCC signaling may be used to polarize cellular activities and orient them to the surrounding environment. That is, netrin causes the direction of DCC activity to stochastically fluctuate. By increasing or decreasing the probability that DCC activity will become oriented to one particular side of the cell, external asymmetric factors orient polarized DCC activity within the cell. The cellular activities regulated by DCC may depend on the cell type and the stage of development.
